# Cotton RLP6 Interacts With NDR1/HIN6 to Enhance Verticillium Wilt Resistance via Altering ROS and SA

**DOI:** 10.1111/mpp.70052

**Published:** 2025-01-22

**Authors:** Dongmei Zhang, Yan Wang, Qishen Gu, Lixia Liu, Zhicheng Wang, Jin Zhang, Chengsheng Meng, Jun Yang, Zixu Zhang, Zhiying Ma, Xingfen Wang, Yan Zhang

**Affiliations:** ^1^ State Key Laboratory of North China Crop Improvement and Regulation, North China Key Laboratory for Crop Germplasm Resources of Education Ministry, Hebei Provincial Key Laboratory of Crop Germplasm Resources Hebei Agricultural University Baoding China

**Keywords:** cotton, eLRR‐RLP6, molecular mechanism, structural variation, Verticillium wilt

## Abstract

Cotton Verticillium wilt (VW) is often a destructive disease that results in significant fibre yield and quality losses in *Gossypium hirsutum* . Transferring the resistance trait of *Gossypium barbadense* to *G. hirsutum* is optional but challenging in traditional breeding due to limited molecular dissections of resistance genes. Here, we discovered a species‐diversified structural variation (SV) in the promoter of receptor‐like protein 6 (RLP6) that caused distinctly higher expression level of *RLP6* in *G. barbadense* with the SV than *G. hirsutum* without the SV. Functional experiments showed that *RLP6* is an important regulator in mediating VW resistance. Overexpressing *RLP6* significantly enhanced resistance and root growth, whereas the opposite phenotype appeared in *RLP6*‐silenced cotton. A series of experiments indicated that RLP6 regulated reactive oxygen species (ROS) and salicylic acid (SA) signalling, which induced diversified defence‐related gene expression with pathogenesis‐related (PR) proteins and cell wall proteins enrichments for resistance improvement. These findings could be valuable for the transfer of the *G. barbadense* SV locus to improve *G. hirsutum* VW resistance in future crop disease resistance breeding.

## Graphical abstract

A species‐diversified structural variation in the RLP6 promoter affected its expression; RLP6 interaction with NDR1/HIN6‐like protein 6 enhanced Verticillium wilt resistance by altering reactive oxygen species and salicylic acid levels.

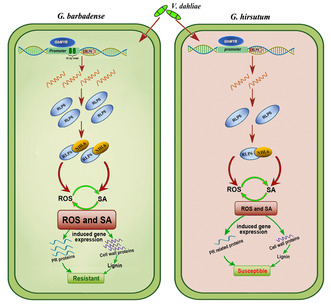

## 1 Introduction

Crop diseases caused by fungi severely limit agricultural productivity, leading to drastic economic losses each year (Song et al. 2020). During pathogen infection, plants have evolved elaborate and effective signalling networks and immune systems for defence against invading pathogen (Robert‐Seilaniantz, Grant, and Jones 2011). Induced immunity begins with pathogen perception, and plants use two major modes of pathogen recognition. In the first mode, immunity is triggered by plant recognition of conserved microbial features termed pathogen‐associated molecular patterns (PAMPs) or host‐derived damage‐associated molecular patterns (DAMPs) by cell surface‐located pattern recognition receptors (PRRs), leading to PAMP‐triggered immunity (PTI; Boller and Felix 2009; Zipfel 2014). Hallmarks of PTI include a cellular influx of calcium, mitogen‐activated protein kinase (MAPK) activation, reactive oxygen species (ROS) burst, accumulation of the defence hormone salicylic acid (SA) and callose deposition, which confer effective resistance against attack by non‐adapted pathogens (Kim et al. 2005; Tsuda et al. 2008; Wang et al. 2020; Couto and Zipfel 2016; Luo et al. 2017; Zhang et al. 2017). In the second mode, intracellular nucleotide‐binding domain leucine‐rich repeat receptors (NLRs) recognise pathogen effector proteins within cells and activate effector‐triggered immunity (ETI; Macho and Zipfel 2015). An effective defence response strongly depends on the action of several plant hormones that ultimately reprogramme the transcriptome. Among them, SA has been identified as one of the key components of the immune signalling in both ETI and PTI (Huang et al. 2020; Lukan and Coll 2022).

ROS production across different subcellular compartments is one of the earliest hallmarks of the plant defence response, which regulates plant immunity (Lukan and Coll 2022). The extracellular ROS burst can act as antimicrobial molecules, strengthen the plant cell wall through oxidative cross‐linking and serves as a local and systemic messenger to induce downstream immune responses (Kadota, Shirasu, and Zipfel 2015; Wang et al. 2014). A suitable equilibrium of ROS is maintained by its chief regulator—SA (Lukan and Coll 2022). The ROS burst not only acts downstream of SA signalling but is also proposed to be a central component of a self‐amplifying loop that regulates SA signalling as well as the balance between different phytohormones (Lukan and Coll 2022). SA is required for the restriction of bacterial, oomycete, fungal and viral pathogens during the hypersensitive response (HR) in various pathosystems (Künstler et al. 2016; Calil and Fontes 2017), such as tobacco mosaic virus and potato virus Y (Chivasa et al. 1997) and *Verticillium dahliae* (Baebler et al. 2014). In Verticillium wilt (VW), SA accumulation is induced by *V. dahliae* infection and is required for PTI in *Arabidopsis* (Zhang et al. 2017; Liu et al. 2014). Enhanced disease susceptibility (*EDS1*), non‐race‐specific disease resistance 1 (*NDR1*), as well as somatic embryo‐genesis receptor kinase (*SERK3/BAK1*) in SA signalling are required in tomato *eLRR‐RLP*‐mediated defence against *V. dahliae* (Fradin et al. 2009, 2011; Wang, Zhou, et al. 2018; Wang, Wang, et al. 2018; Wang, Cheng, et al. 2018). Our previous studies also demonstrated that SA and H_2_O_2_ contribute to VW resistance in cotton (Zhang et al. 2017; Mo et al. 2021). Yet knowledge of the exact functional genes that integrate SA and ROS signalling in the cotton defence response is still limited.

PAMPs are perceived by PRRs in the plasma membrane. The PRRs in plants are either receptor‐like kinases (RLKs) or receptor‐like proteins (RLPs; Couto and Zipfel 2016; Luo et al. 2017). RLPs are cell surface receptors that typically consist of an extracellular leucine‐rich repeat domain, a transmembrane domain and a short cytoplasmatic tail and share structural similarity with RLKs but lack a cytoplasmic kinase domain (Fritz‐Laylin et al. 2005). This likens them to the toll‐like receptors (TLRs) involved in mammalian immunity, which also contain an extracellular LRR domain and a short cytoplasmic tail (Medzhitov, Preston‐Hurlburt, and Janeway Jr 1997; Jamieson, Shan, and He 2018). Several plant RLPs have been found to play critical roles in disease resistance, such as the tomato Cf‐9 (Jones et al. 1994), Ve (Kawchuk et al. 2001) and the apple HcrVf2 proteins (Belfanti et al. 2004), which mediate resistance against the fungal pathogens *Cladosporium fulvum*, *Verticillium* spp. and *Venturia inaequalis*, respectively. In cotton, several *RLP* genes such as *Ve1*, *Gbve1* (Zhang et al. 2012), *Gbvdr3* (Chen et al. 2016), *Gbvdr5* (Yang, Zhang, et al. 2014) and *GbVe* (Zhang et al. 2011) have been proved to play roles in VW resistance; however, the downstream signalling mechanism leading to the restriction of the pathogen is still unclear.

*Gossypium barbadense* is a cultivated tetraploid, grown for its exceptionally high‐quality fibres and high resistance to *V. dahliae*. To improve the disease resistance of *Gossypium hirsutum* , which is planted over 90% of the cotton acreage worldwide, a proposed approach is to transfer superior related traits from *G. barbadense* into *G. hirsutum* (Ma et al. 2021). This option is very challenging using traditional breeding due to limited molecular dissections of resistance genes. Here, we identified a structural variation (SV) harboured by the promoter of an *RLP* gene (*GbM_D09G2012*) using a modern cotton genome assembly (Ma et al. 2021) and comparison between *G. hirsutum* and *G. barbadense* . We investigated the SV effect on the physiological basis and molecular mechanism underlying Verticillium wilt and demonstrated that the SV*‐GbRLP6* pair is crucial to VW resistance of *G. barbadense* and could be used for *G. hirsutum* improvement.

## 2 Results

### 2.1 RLP6 Promoter Polymorphism Is Associated With Cotton VW Resistance

**FIGURE 1:**
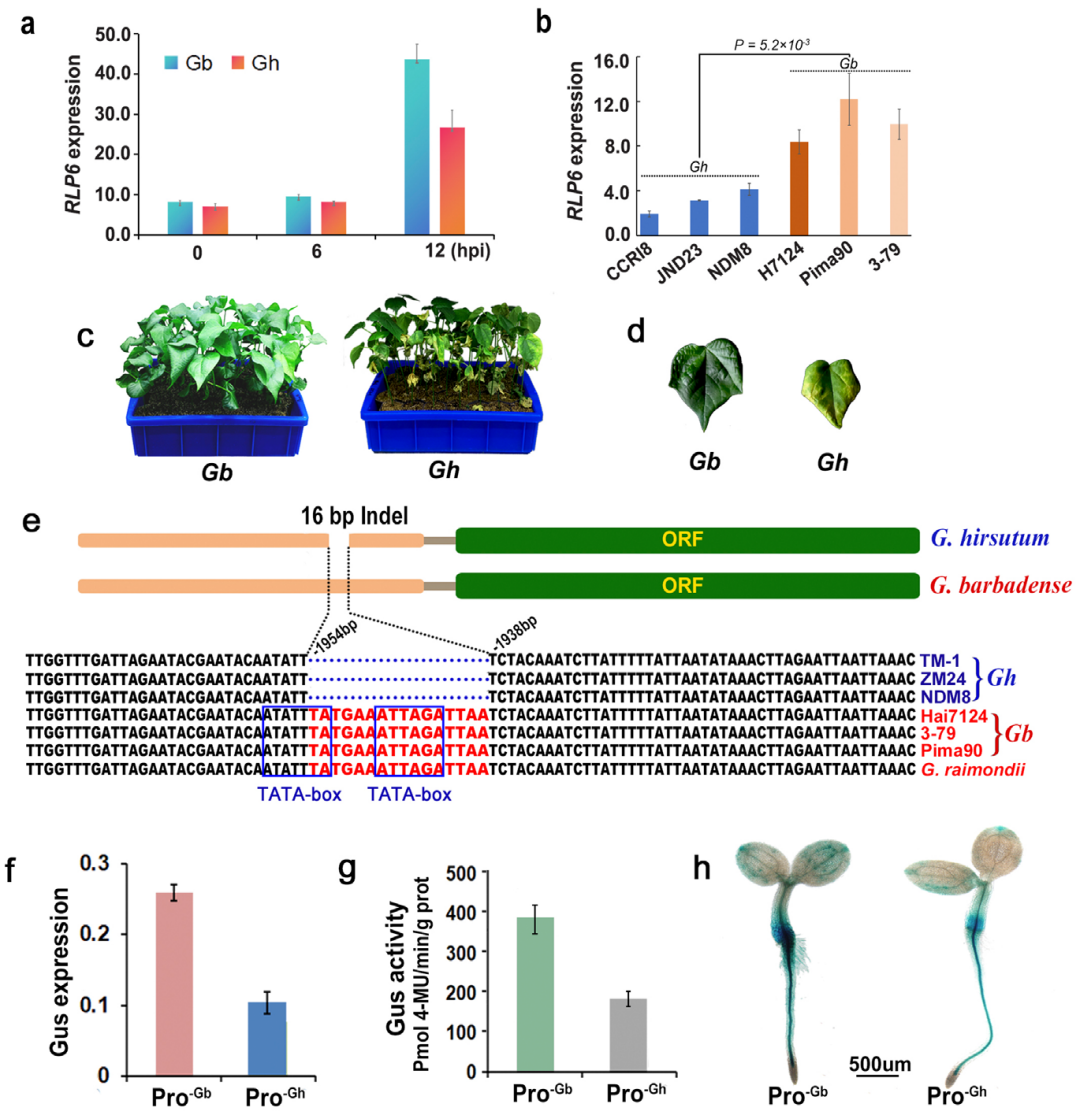
*RLP6* promoter polymorphism influenced the gene expression level. (a) Reverse transcription‐quantitative PCR (RT‐qPCR) analysis of *RLP6* expression in *Gossypium barbadense* ‘Pima90’ (Gb) and *Gossypium hirsutum* ‘CCRI8’ (Gh) inoculated with strongly pathogenic *Verticillium dahliae* LX2‐1 with 7‐day old seedlings. (b) RT‐qPCR analysis of *RLP6* in *G. barbadense* ‘Pima90’, ‘3–79’, ‘H7124’ and *G. hirsutum* ‘CCRI8’, ‘JND23’ and ‘NDM8’ under *V. dahliae* challenge at 12 h post‐inoculation (hpi). *GhUBQ14* was used as an internal control. Data are means (±*SE*) of three biological repeats. (c, d) The disease symptoms of *G. barbadense* (Pima90) and *G. hirsutum* (CCRI8) infected with *V. dahliae* LX2‐1 in the greenhouse. (e) Sequence analysis of the polymorphic *RLP6* promoter region across different cotton species based on the published assemblies. (f) RT‐qPCR analysis the *GUS* gene expression levels of transgenic *Arabidopsis* at 4 days post‐inoculation, driven by the *GbRLP6* promoter (Pro^−Gb^) region or *GhRLP6* promoter (Pro^−Gh^) region. The *Arabidopsis Actin* gene was used as an internal control. Data are means (±*SD*) of three biological repeats. (g) Quantitative determination of β‐glucuronidase (GUS) activity in the two types of transgenic *Arabidopsis* of 20‐day‐old seedlings. (h) GUS histochemical staining analysis reflected different GUS activity in the two types of transgenic *Arabidopsis* plants.

In our recent study, a number of SV gene pairs were detected between the *G. barbadense* and *G. hirsutum* genomes (Ma et al. 2021). Here, we focused on a single‐copy gene, *RLP6*, located on chromosome Dt09. We found it was induced by *V. dahliae* inoculation in both *G. barbadense* and *G. hirsutum* (Figure 1a), with higher expression in *G. barbadense* ‘Pima90’ and stronger VW resistance (Figure 1b,c) than in *G. hirsutum* ‘CCRI8’, ‘JND23’ and ‘NDM8’ (Figure 1b,d). However, the 100% sequence identity of the gene between the two species encouraged us to investigate if sequence variations existed in the promoter, which is widely recognised as a potential causal element for downstream gene expression diversity. We discovered an SV in the promoter, with a 16‐bp insertion in the promoters of *RLP6* genes of *G. barbadense* accessions but a same‐sized deletion in those of *G. hirsutum* cultivars. Phylogenetic analysis of the ancestral diploid species *Gossypium raimondii* showed the same sequence variation as *G. barbadense* (Figure 1e), an indicator of species‐diversified SV occurrence during the evolution of the two cultivated tetraploid cottons. To test if the SV affected the power of the promoter activation, we produced transgenic *Arabidopsis* lines with the two sorts of promoters from the two cotton species. *Arabidopsis* with β‐glucuronidase (*GUS*) driven by the *G. barbadense* promoter showed a much higher *GUS* expression level and enzyme activity than when driven by the *G. hirsutum* promoter (Figure 1f–h), indicating that the *G. barbadense* promoter conferred higher expression of *RLP6*. We also transferred the *RLP6* gene driven by its own promoter from *G. barbadense* (GbPro‐RLP6) or *G. hirsutum* (GhPro‐RLP6) into *Arabidopsis*. When the transgenic plants were inoculated with *V. dahliae*, we found that the *GbPro‐RLP6* transgenic *Arabidopsis* displayed obvious improved VW resistance compared to the *GhPro‐RLP6* transgenic plants (Figure S1), indicating that *RLP6* promoter polymorphism is associated with VW resistance. These findings suggest that the genomic SV and cognate gene *RLP6* might play a prominent role and is reminiscent of a potential utility in *G. hirsutum* improvement that will be addressed below.

### 2.2 Altered Expression of *RLP6* Significantly Affects VW Resistance and Root Biomass

**FIGURE 2:**
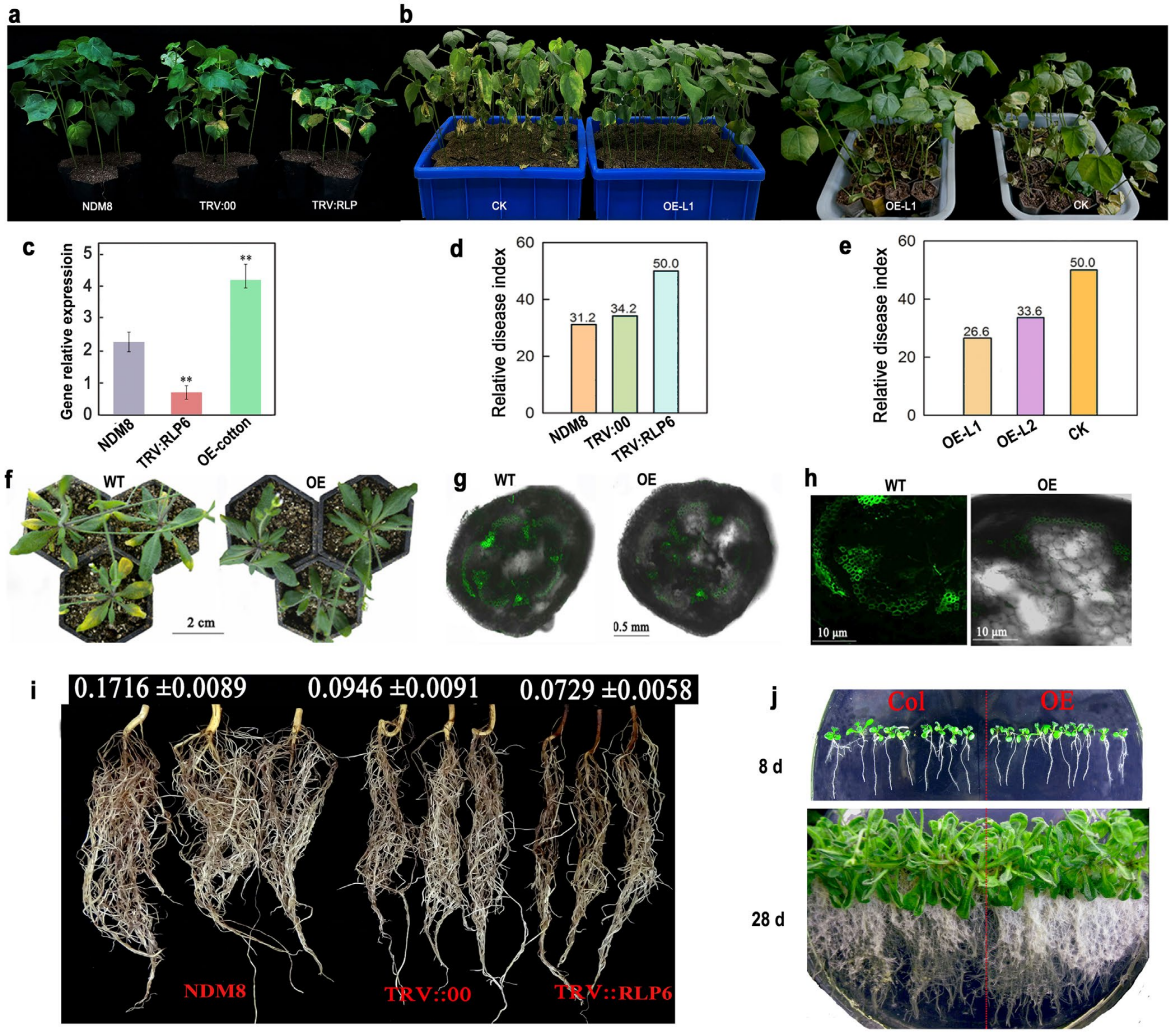
Functional analysis of *RLP6* gene via gene silencing and overexpression assays. (a) Silencing of *RLP6* gene in tolerant variety NDM8 led to obviously decreased resistance compared with the mock (TRV:00). (b) Representative images of wild‐type (CCRI8) and different overexpression (OE) plants under *Verticillium dahliae* challenge. CK, negative control. (c) The expression level of *RLP6* in the virus‐induced gene silencing (VIGS) and overexpression cotton. (d, e) The disease index (DI) was calculated from the disease level of at least 35 independent seedlings. The DI of CCRI8 and OE transgenic lines, 35 cotton seedlings were used for Verticillium wilt disease resistance detection. (f) *RLP6* overexpression in *Arabidopsis* made transgenic plants highly resistant compared with the wild type. (g, h) Infection process at 14 days post‐inoculation, based on green fluorescent protein (GFP)‐tagged *V. dahliae*, in wild‐type (WT) and *RLP6*‐overexpressing *Arabidopsis*. (i, j) Influence of *RLP6* on plant root growth. The root amount was markedly decreased when suppressing *RLP6* expression via virus‐induced gene silencing, and this result was also demonstrated in *RLP6*‐overexpressing *Arabidopsis*.

To ascertain the role of *RLP6* in VW resistance, we constructed a virus‐induced gene silencing (VIGS) vector targeting a motif in *RLP6,* as it exists in a single copy in the cotton genome, and successfully silenced the gene in the VW‐tolerant *G. hirsutum* 'NDM8'. The *RLP6* transcript level was significantly reduced in the silenced cotton compared to the control plant. Silencing *RLP6* in cotton resulted in compromised resistance, making the tolerant NDM8 susceptible, suggesting that *RLP6* positively regulates VW resistance (Figure 2a–c). Furthermore, we overexpressed *RLP6* in the susceptible cotton cultivar CCRI8 and *Arabidopsis* (Col) to examine the role of *RLP6* in VW resistance. The transgenic cotton was confirmed by PCR and Sanger sequencing (Figure S2). We found that the transgenic lines displayed higher expression levels of *RLP6* and improved resistance to *V. dahliae* compared with the control (Figure 2c,e–h). Moreover, we found that *RLP6* gene affected root development in both transgenic *Arabidopsis* and VIGS cotton, which is similar to VW resistance enhancement in tomato (Nazar et al. 2018). As shown in Figure 2i, the *RLP6‐*silenced cotton displayed a reduced amount of roots, with an average 22.9% decrease compared to the control (TRV:00). The converse was true in the transgenic *Arabidopsis* seedlings (Figure 2j). These results proved that the alteration of VW resistance and root biomass were caused by the expression changes of *RLP6*.

### 2.3 Altering *RLP6* Expression Influenced H_2_O_2_ , SA and Lignin Accumulation

**FIGURE 3:**
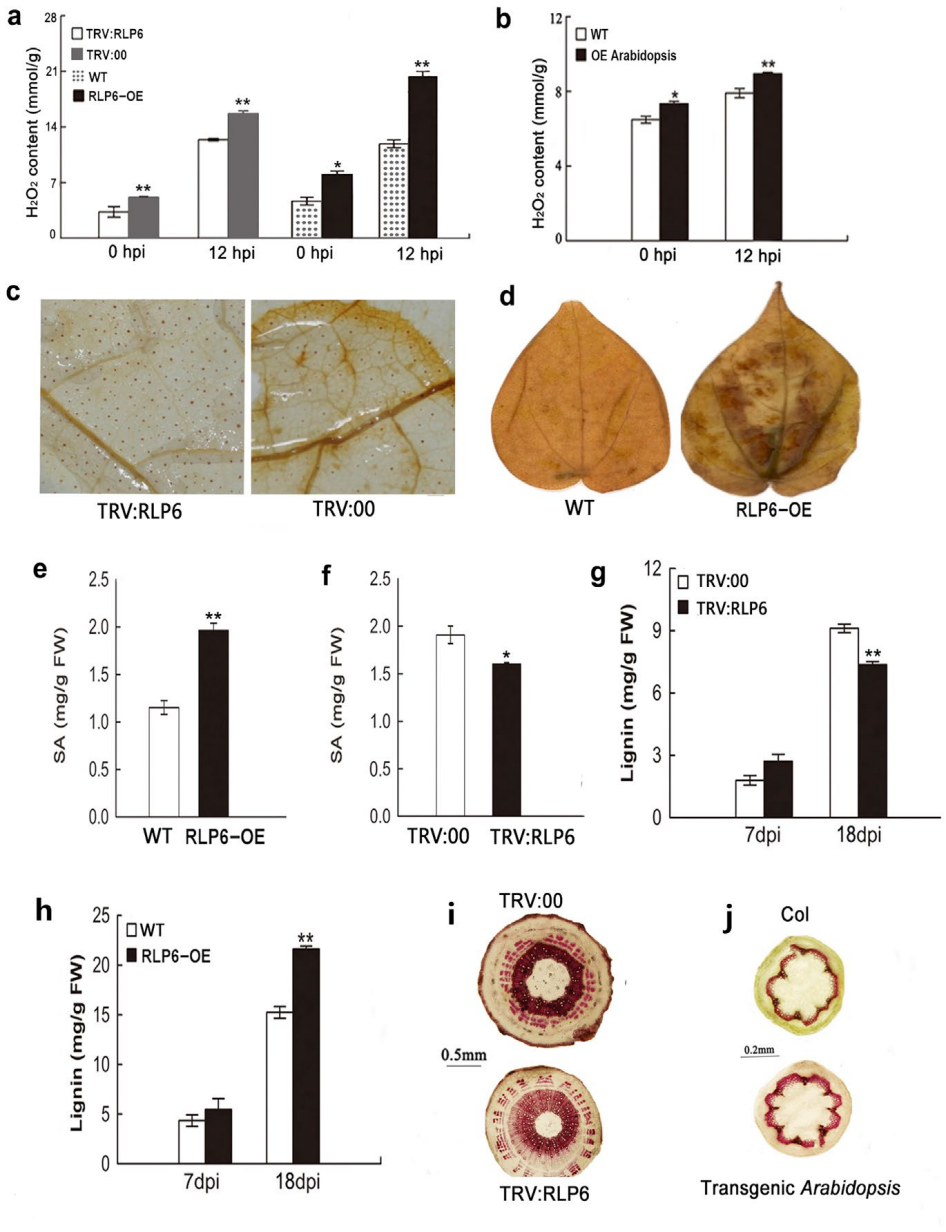
Detection of H_2_O_2_, salicylic acid (SA) and lignin. The H_2_O_2_ contents in virus‐induced gene silencing (VIGS) cotton, overexpressing (OE) cotton (a) and OE *Arabidopsis* (b). The bars represent means ± *SD* of three biological replicates. hpi, hours post‐inoculation. 3,3′‐diaminobenzidine (DAB) staining of H_2_O_2_ accumulation in the silenced cotton (c) and the OE cotton (d). (e) SA contents in leaves from OE cotton with 25‐day‐old seedling (using 10‐day‐old seedling inoculated by *Verticillium dahliae*, at 15 days post‐inoculation (dpi) the infected seedling was harvested for SA detection). (f) SA contents in leaves from VIGS cotton with 40‐day‐old seedling (the 10‐day‐old seedling was infiltrated with *Agrobacterium* for *RLP6* silencing. At about 15 days post‐infiltration, the silenced cotton was inoculated with *V. dahliae*, at 15 dpi the infected seedling was also harvested for SA detection). (g, h) The lignin content in the stem from OE‐ and VIGS cotton (g) and OE cotton (h) under *V. dahliae* challenge. Lignin contents determined as lignin thioglycolic acid equivalents (milligrams per gram of dry weight). Quantifications were performed using alkaline lignin as standard. Each value represents the mean ± *SD* of three biological replicates. The asterisks indicated statistically significant differences, as determined by Student's *t* test (*p < 0.05, **p < 0.01). (i, j) Light microscopy image of hypocotyl cross‐sections at 18 dpi after staining with phloroglucinol‐HCl to detect lignin in VIGS cotton (i) and *RLP6*‐overexpressing *Arabidopsis* (j).

Based on the fact that ROS are produced upon pathogen perception (Luo et al. 2017), we examined H_2_O_2_ levels in fresh leaves and discovered that the H_2_O_2_ levels were significantly increased in the transgenic cotton and *Arabidopsis* overexpressing *RLP6*, whereas they were decreased in VIGS cotton (Figure 3a,b). Staining of the leaf cells with 3,3′‐diaminobenzidine (DAB) for H_2_O_2_ also revealed that the *RLP6*‐silenced cotton produced much lower amounts of H_2_O_2_ than TRV:00, resulting in darker staining (Figure 3c), whereas the *RLP6*‐overexpressing cotton produced much higher amounts (Figure 3d). These results demonstrated that altering *RLP6* expression could influence the H_2_O_2_ level. To determine the effect of *RLP6* on SA levels, the free SA contents in the *RLP6*‐silenced cotton and transgenic overexpression cotton with the corresponding control were investigated. The free SA levels were significantly decreased in the *RLP6*‐silenced cotton, whereas increased in the *RLP6*‐overexpressing cotton (Figure 3e,f). When *RLP6* was silenced in cotton, the lignin content was remarkedly reduced compared to the control (TRV:00) (Figure 3g) and increased when *RLP6* was overexpressed in cotton (Figure 3g,h). A similar result was also demonstrated by histochemical analysis of lignin deposition in stem cross‐sections of the silenced cotton and transgenic *Arabidopsis* lines (Figure 3i,j). These results indicated that *RLP6* influenced the biosynthesis of H_2_O_2_, SA and lignin in plants when challenged by the fungus.

**FIGURE 4:**
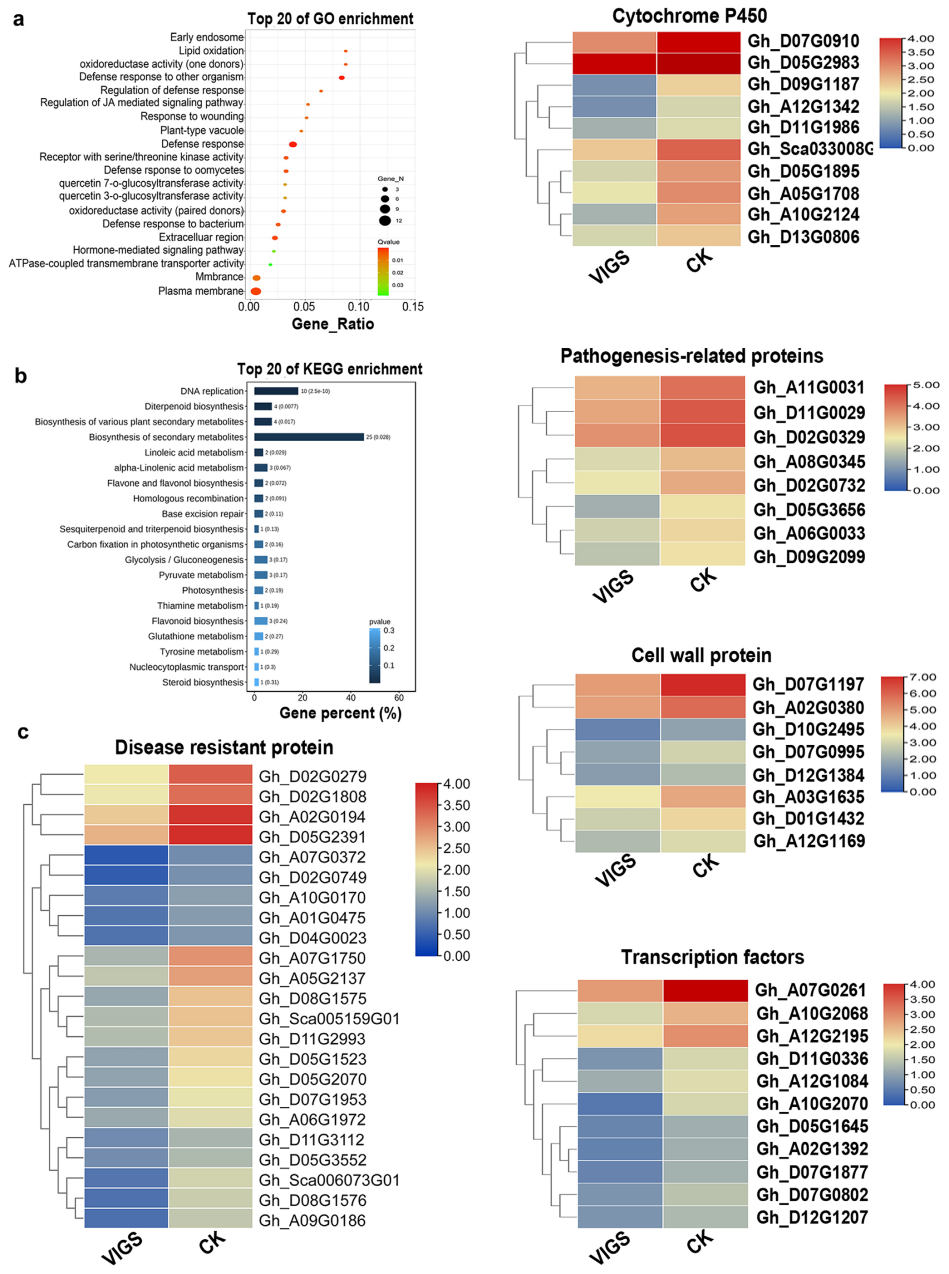
Transcriptome profiles of genes in *RLP6*‐silenced cotton. (a) Enrichment analysis of downregulated genes. The top 20 significantly enriched biological process GO terms are shown. (b) KEGG enrichment analysis. (c) Heat map of the defence‐related genes (*p* < 0.05, log_2_fold‐change > |2|). VIGS, virus‐induced gene silencing; CK, negative control.

We used RNA‐Seq to identify the signalling pathways regulated by RLP6 in response to *V. dahliae*. Of all the differentially expressed genes (DEGs), 443 downregulated DEGs were identified in the gene‐silenced cotton. Gene ontology (GO) analysis showed that the ‘defence response’ metabolic process was highly enriched (Figure 4a,b). In detail, 23 disease resistant proteins, 10 cytochrome P450, 8 pathogenesis‐related (PR) proteins, 8 cell wall proteins and 11 transcription factors were enriched (Figure 4c). The expression of several genes selected from these DEGs was also confirmed by reverse transcription‐quantitative PCR (RT‐qPCR) analysis in silenced and transgenic cotton plants (Figure S2). These results suggested that knock‐down of *RLP6* impairs signal transduction and/or signal amplification that diminishes cell wall fortification, as reflected by the different H_2_O_2_, SA and lignin content in silenced and overexpression plants.

### 2.4 RLP6 Directly Interacted With NDR1/HIN6‐Like Protein 6

**FIGURE 5:**
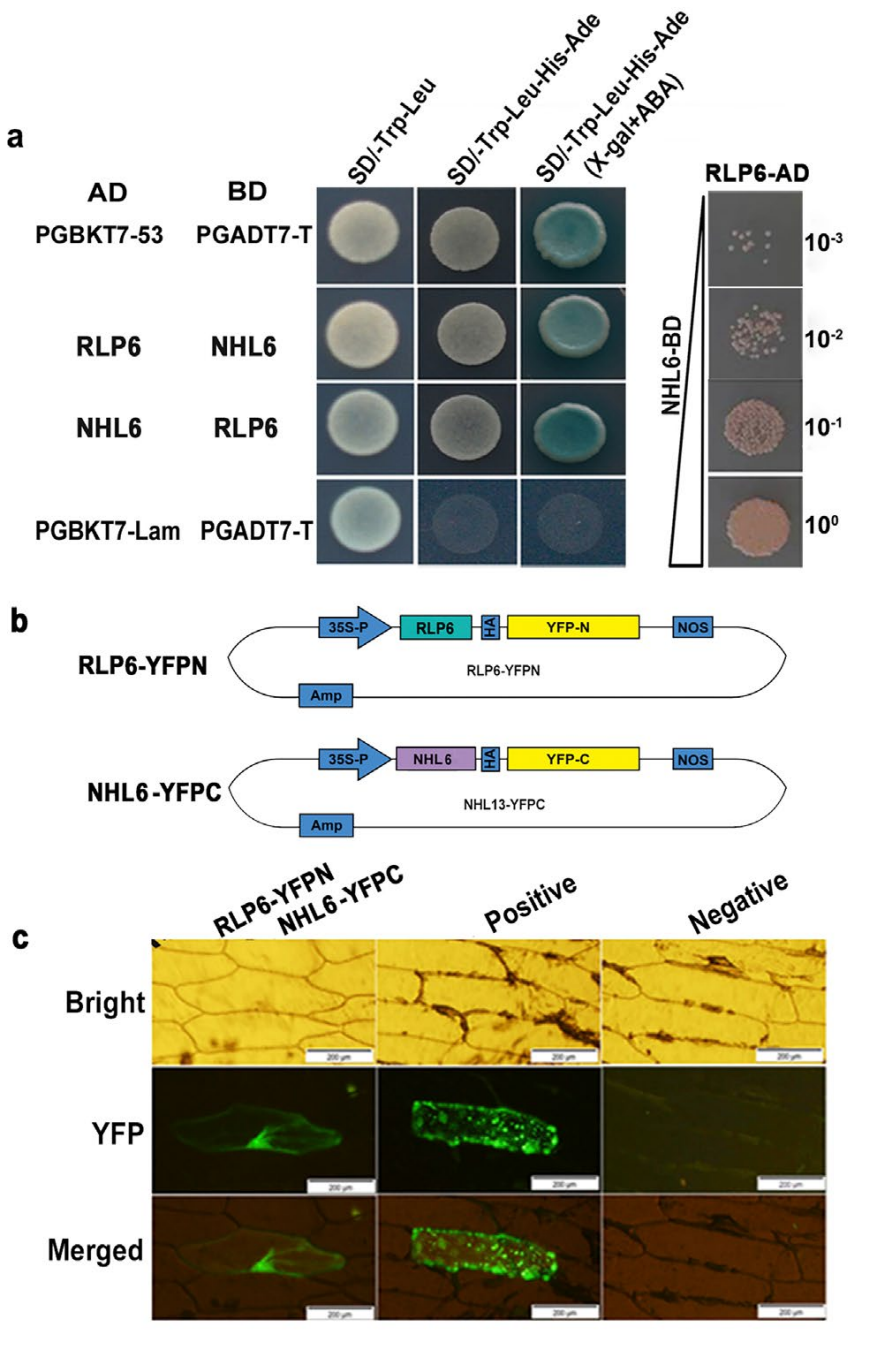
RLP6 interacts with NHL6. (a) Yeast two‐hybrid assay showed the interaction between RLP6 and NHL6. (b, c) Vector construction (b) and bimolecular fluorescence complementation analysis (c) of RLP6 and NHL6 molecular interaction in onion epidermal cells (scale bars = 200 μm). The negative control was transformed with empty vector, and the positive control was co‐transformed with Nodulin‐35 and AtTRX‐o1.

To explore the target of RLP6, we screened RLP6‐interacting proteins using a yeast two‐hybrid (Y2H) assay with a cDNA library prepared from cotton challenged with *V. dahliae*. Among the potential interacting genes (Figure S3), one gene, *GhM_A05G3087*, was particularly interesting as it encodes an NDR1/HIN‐like protein 6 (NHL6) in the SA pathway and covered by DEGs. RLP6 and NHL6 passed the toxicity testing and autoactivation identification of the bait yeast (Figure S4). Then the interaction between RLP6 and NHL6 was confirmed by the Y2H one‐on‐one interaction testing (Figure 5a,b). Interaction fluorescence signals were also observed in onion epidermal cells when RLP6‐YFPN and NHL6‐YFPC were transformed together, while the interaction of Nodulin‐35 and AtTRX‐o1 was used as a positive control (Figure 5b,c). These results demonstrate that RLP6 could directly interact with NHL6 in vivo and in vitro.

**FIGURE 6:**
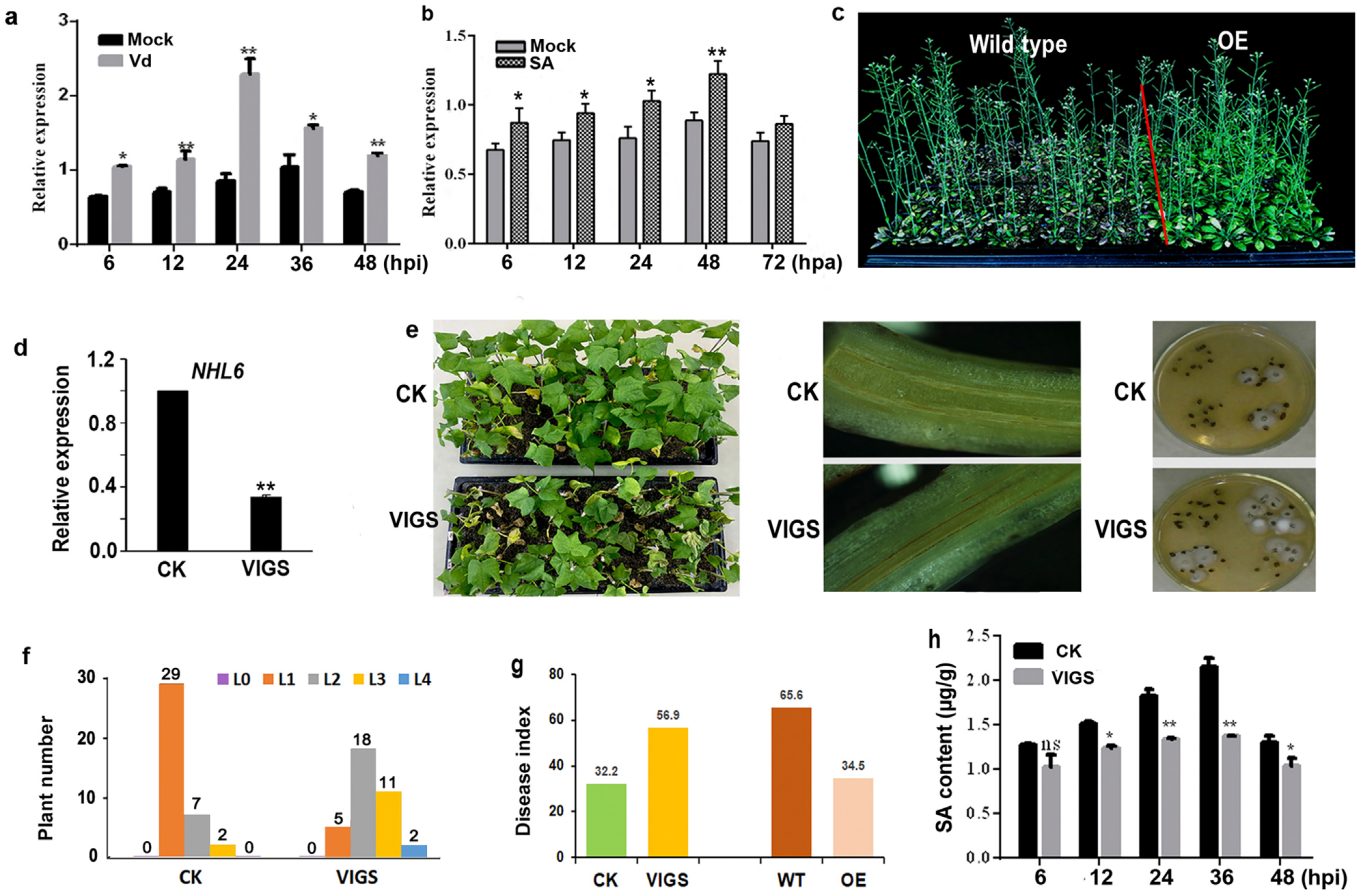
Functional analysis of *NHL6* gene via gene expression, silencing and overexpression assays. (a, b) Reverse transcription‐quantitative PCR analysis of *NHL6* gene expression under *Verticullium dahliae* (a) and SA treatments (b). hpi, hours post‐inoculation. (c) Validation of the function of *NHL6* by ectopic expression in *Arabidopsis*. Three‐week‐old plants were inoculated with LX2‐1 (5 × 10^7^ conidia/mL). Transgenic *Arabidopsis* (OE) showed obvious improved resistance at 20 days post‐inoculation (dpi). (d) The efficiency of *NHL6* gene under our experimental conditions when the *CLA1* was successfully silenced. (e) The disease resistance performance of silencing *NHL6* in tolerant (NDM8) cultivar at 20 dpi. Tissue browning was rare in vascular bundles from control (CK) plants, whereas severe in longitudinal sections from silenced (VIGS) plants. Twenty days after *V. dahliae* inoculation, surface‐sterilised hypocotyl sections prepared from CK and silenced plants were placed on agar medium at 7 dpi. The number of stem sections from which fungus grew represented the extent of fungal colonisation. (f, g) The rate of diseased plants (f) and disease index (g) were measured at 20 dpi. (h) Salicylic acid (SA) contents in leaves from CK and silenced plants. Significant difference, ns, *p* > 0.05, *p < 0.05, **p < 0.01.

We further investigated *NHL6* gene function via expression analysis, overexpression and VIGS. The results indicated that the expression of *NHL6* was significantly upregulated at 6 h post‐inoculation (hpi) and reached a peak at 24 hpi (Figure 6a). Based on our previous reports that cotton SA level increased after inoculation with *V. dahliae* (Zhang et al. 2017; Mo et al. 2021), we asked how expression of *NHL6* responded to SA treatment. We found that the transcript level of *NHL6* in cotton was significantly increased under SA treatment (*p* < 0.05; Figure 6b). *Arabidopsis* with *NHL6* gene overexpression exhibited enhanced VW resistance, changing the disease resistance level from susceptible (disease index [DI] 62.3) to tolerant (DI 27.8; Figure 6c). We also investigated the function of *NHL6* in defence against *V. dahliae* infection via VIGS. As shown in Figure 6d, the newly developing tissues of leaf and stem displayed an albino phenotype in the cotton infiltrated with agrobacteria carrying *CLA1*, confirming that the VIGS system worked efficiently. At the same time, we checked the expression of *NHL6* and found that its expression was significantly reduced in the *NHL6*‐VIGS cotton (Figure 6d). When plants were inoculated with *V. dahliae*, the *NHL6‐*silenced cotton was more susceptible (Figure 6f,g). Furthermore, the SA level markedly decreased in silenced cotton, indicating *NHL6* is a positive regulator of the SA‐mediated plant defence response (Figure 6h). These results demonstrate that the expression of *NHL6* is involved in plant VW resistance, in accordance with *RLP6*.

We performed dual silencing of *RLP6‐NHL6* in cotton. We found that both *RLP6* and *NHL6* displayed similar expressions. When the *RLP6* gene was silenced, the expression of *NHL6* decreased with the increase of *RLP6* silencing efficiency, whereas when the *NHL6* gene was silenced, *RLP6* expression did not change significantly with the increase of *NHL6* silencing efficiency. At about 12 days post‐infiltration, we measured *RLP6* and *NHL6* transcription and found that they were both significantly suppressed compared to the control (CK) plants. Subsequently, the dual‐silenced *RLP6*‐*NHL6* plants were inoculated with the highly aggressive defoliating *V. dahliae* strain LX2‐1. At 20 dpi, the *RLP6*‐*NHL6*‐silenced cotton displayed more serious symptoms of VW than the *RLP6*‐silenced cotton (Figure S5). These results reflect the cooperation between RLP6 and NHL6 in plant defence.

### 2.5 MYB308 Directly Bound the Promoter of *RLP6* and Regulated Its Transcription

**FIGURE 7:**
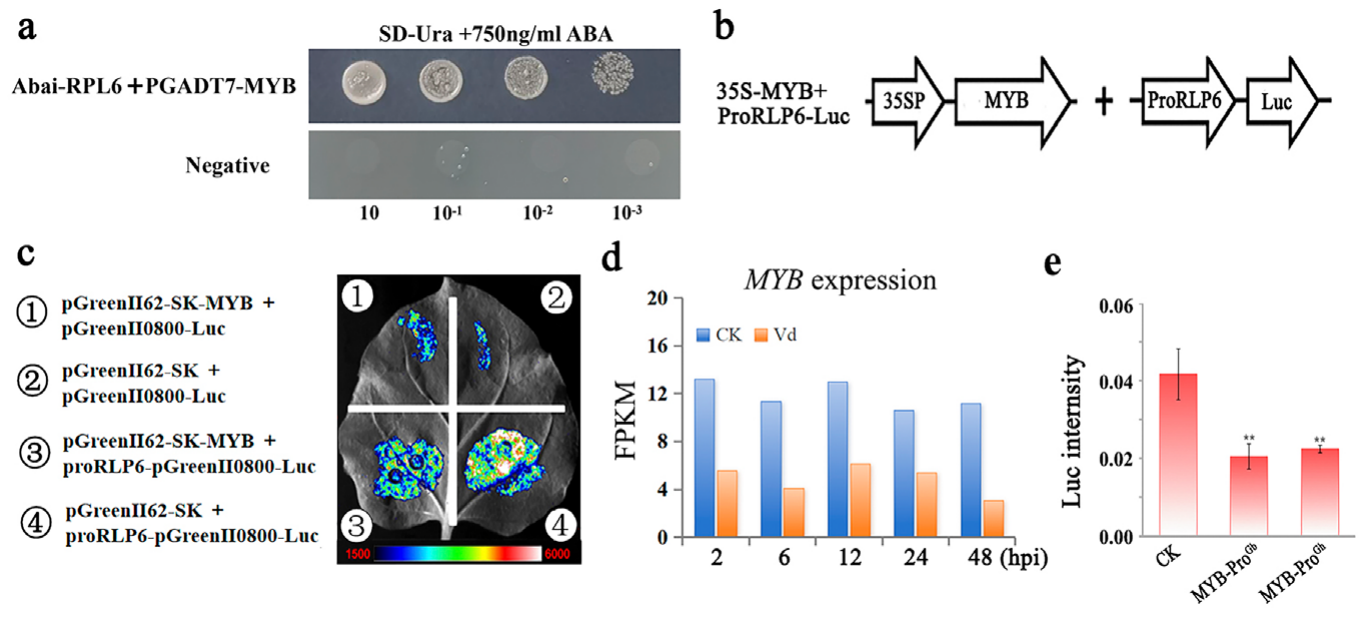
MYB308 directly bound the promoter of *RLP6* and regulated its transcription. (a) Yeast one‐hybrid analysis of the interaction of MYB308 and the *RLP6* promoter. (b) The *MYB308* gene and *RLP6* promoter information in the chart. (c) Transcription activity of *MYB308* was tested in *Nicotiana benthamiana* leaves using a luciferase (Lu)‐based system. *Agrobacterium tumefaciens* GV3101 harbouring different constructs was infiltrated into different leaf regions. (d) *MYB308* expression heat map upon *Verticillium dahliae* challenge based on RNA‐Seq data. CK, negative control; hpi, hours post‐inoculation. (e) Transient expression assay for quantification of promoter activity by MYB308. Significant difference, **p < 0.01.

To identify the corresponding transcription factor binding to the *RLP6* promoter, we investigated the potential transcription factor by yeast one‐hybrid (Y1H) screening and one‐to‐one validation. The cotton MYB308 transcription factors were inserted into pGADT7 vectors. As shown in Figure 7a, only the proRLP6::pAbAi Y1H strain that positively expressed cotton *GhMYB308* transcription factors was able to grow on SD/−Leu medium supplemented with 700 ng/mL aureobasidin A (Figure 7a). To verify whether MYB308 could directly bind to the promoter region of *RLP6* and regulate its expression, we performed a transactivation assay. The results revealed that 35S‐MYB308 significantly suppressed *RLP6* expression (Figure 7b,c). We also found that *GhMYB308* displayed obvious decreased expression upon *V. dahliae* infection (Figure 7d). Thus, GhMYB308 can directly bind to the promoter of the *RLP6* gene and regulate its expression.

Having determined that MYB308 binds to the promoter of RLP6 (*p*RLP6), we further investigated the difference of *GbRLP6* and *GhRLP6* promoter activity by transcription factor MYB308. A double reporter plasmid containing the firefly luciferase (Luc) driven by *p*RLP6^Gb^ or *p*RLP6^Gh^ (about 1.5 kb from the transcription start site) and the Renilla luciferase (Re) driven by the CaMV 35S promoter, together with an effector plasmid expressing MYB308 was co‐transformed into *Nicotiana benthamiana* leaves (Hellens et al. 2005). Analysis of infiltrated *N. benthamiana* tissues revealed that MYB308 repressed the Luc signal in both *p*RLP6^Gh^ and *p*RLP6^Gb^ compared to the negative control, while there was no significant difference between *p*RLP6^Gh^ and *p*RLP6^Gb^ (Figure 7e). However, we found that the MYB308 expression was significantly decreased upon *V. dahliae* challenge, and its expression level was lower in *G. barbadense* than in *G. hirsutum* , which finally led to the relatively higher expression level of RLP6 in *G. barbadense* than in *G. hirsutum* .

### 2.6 The Species‐Diversified SV Can Be Used in *G. hirsutum* VW Resistance Improvement

**FIGURE 8:**
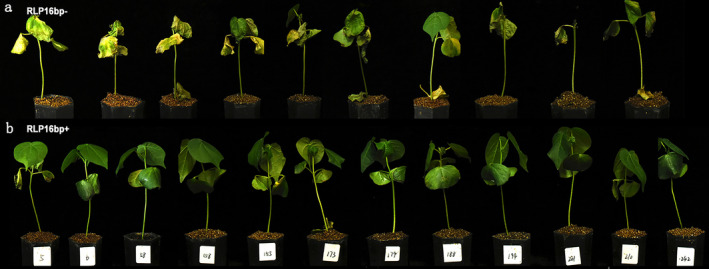
Disease resistance of the two different genotypes of introgression lines derived from a Pima90 × CCRI8 cross. The individuals with RLP6^16bp+^ genotype grew robustly, exhibiting distinctly improved Verticillium wilt resistance (a) compared to those with RLP6^16bp‐^ genotype (b) at 14 days post‐inoculation.

To potentially and effectively use the genomic variation of *G. barbadense* in *G. hirsutum* breeding programmes, a population of 296 stably inherited introgression lines was generated from a cross between the susceptible cultivar *G. hirsutum* 'CCRI8' and the highly resistant cultivar *G. barbadense* 'Pima90'. First, the parents were genotyped via the SV‐derived marker RLP6^16bp‐indel^, with CCRI8 as RLP6^16bp‐^ and Pima90 as RLP6^16bp+^. Then population genotyping was conducted using the RLP6^16bp‐indel^ marker and the VW resistance was evaluated in an environmentally controlled growth chamber. Among the 296 individual lines tested, 12 were genotyped as RLP6^16bp+^ and the others as RLP6^16bp‐^. All the lines with RLP6^16bp+^ grew robustly, exhibiting distinctly improved VW resistance compared to CCRI8 at 20 dpi while the individuals with RLP6^16bp‐^ were severely diseased (Figure 8a,b). The results highlight the utility of the SV and cognate gene *RLP6* of *G. barbadense* as germplasm in *G. hirsutum* resistance breeding.

## 3 Discussion

In the present work, we characterised an eLRR‐RLP gene *RLP6* with a VW resistance function and its underlying molecular mechanism in cotton. *RLP6* obviously differed from the other reported eLRR‐RLP genes *GbVe1* (Zhang et al. 2012), *Gbvdr3* (Chen et al. 2016) and *Gbvdr5* (Yang, Zhang, et al. 2014), with relatively low sequence identity of 69.5%, 65.8% and 64.4% compared to *RLP6*, respectively (Figure S6). *RLP6* was physically located in the D09 chromosome with only a single copy in the whole genome, which was different from the three other reported eLRR‐RLP genes, which are present in multiple copies in the A01 (C1) and D01 (c15) chromosomes. According to the features of signal peptide, LRRs (extracellular domain), transmembrane domain and short intracellular domain, we inferred that *RLP6* might play a role in signal transmission during the process of resisting *V. dahliae*.

Plant hormones play significant roles in regulating developmental processes and signalling networks involved in biotic and abiotic stresses (Durrant and Dong 2004; Bari and Jones 2009). The crucial role of SA as an immune signal has been widely reported in basal defence and the amplification of local immune responses (Vlot, Dempsey, and Klessig 2009; Zheng et al. 2015). For example, exogenous treatment with SA activates various PR protein gene expressions, induces resistance to multiple pathogens, triggers HR and orchestrates systemic acquired resistance (SAR) establishment (Durrant and Dong 2004; Ding and Ding 2020; Vlot et al. 2021; Kachroo, Liu, and Kachroo 2020). The pathogen‐induced SA accumulation generated from the isochorismate pathway in chloroplasts and/or from cinnamate produced by phenylalanine ammonia‐lyase further triggers extensive transcriptional reprogramming, leading to enhanced plant resistance (Bernsdorff et al. 2016; Mo et al. 2021). SA can also mediate plant defence via regulation of plasmodesmata permeability in a callose‐dependent manner during virus spreading (Wang et al. 2013; Huang et al. 2020). Our previous research indicated that *V. dahliae* infection could also induce SA accumulation and hence heightened cotton VW resistance (Zhang et al. 2017; Mo et al. 2021); however, the underlying mechanism is not fully understood. In this study, the results of significantly higher SA level in transgenic cotton than in wild‐type plants, as well as significantly decreased SA content in the silenced cotton, demonstrated involvement of *RLP6* in the SA signalling pathway in cotton. Thus, we deduced that *RLP6* possibly served as a connecting link between PTI and SA signalling activation in cotton.

More interestingly, we found that *RLP6* induced *RbohD* expression (data not shown). *RhobD* is one of the key components of PTI, responsible for producing the majority of extracellular ROS upon pathogen infection (Kadota, Shirasu, and Zipfel 2015; Li et al. 2021). H_2_O_2_ can induce SA accumulation (Dubiella et al. 2013; Luo et al. 2017), and in turn SA application also induces H_2_O_2_ production and cell death (Yang et al. 2013; Wang, Zhou, et al. 2018; Wang, Wang, et al. 2018; Wang, Cheng, et al. 2018), demonstrating that H_2_O_2_–SA forms a positive feedback, which propagates a mutual activation circuit to magnify disease signalling and is essential for cell death induction and SAR. We also observed that both H_2_O_2_ and SA levels increased when overexpressing *RLP6*, implying that H_2_O_2_–SA positive feedback also positively mediated VW resistance. Also, previous research proved that H_2_O_2_ production relies on RbohD activation, probably through phosphorylation by BIK1 and CPKs protein kinases (Dubiella et al. 2013; Kadota, Shirasu, and Zipfel 2015; Li et al. 2014). How RLP6 activates *RbohD* expression needs to be further investigated.

During pathogen infection in host, *V. dahliae* could secrete more than 700 putative effectors that are thought to function in the host apoplast to promote disease (Klosterman et al. 2009). Emerging evidence has shown that pathogen virulence effectors can also target SA signalling to attenuate SA accumulation of the host during pathogen attack, resulting in inhibition of host defence responses. For example, *Phytophthora sojae* successfully disrupts the SA metabolism pathway of host plant via its *ISC* effectors (Qin et al. 2022). The *Arabidopsis* downy mildew pathogen *Hyaloperonospora arabidopsidis* secretes the effector HaRxL44 to degrade the positive regulator of plant immunity and to attenuate SA‐dependent gene expression, leading to enhanced host susceptibility (Caillaud et al. 2013). Similarly, the bacterial pathogen *Xanthomonas campestris* pv. *vesicatoria* secretes the type III effector protein XopJ to interfere with SA‐dependent defence responses by inhibiting the proteasome in pepper (Üstün, Bartetzko, and Börnke 2013). These indicate that SA signalling is one of the major virulence targets of pathogen effectors. In the present work, we revealed that cotton overexpressing *RLP6* possibly overcame the virulence target of *V. dahliae* effectors to the SA pathway, and activated the SA signal, and thereby promoted root growth to successfully defeat pathogen infection.

In the present study, the *RLP6*‐silenced cotton displayed reduced root amount, indicating that *RLP6* may be in a hub of VW resistance and root biomass. Signalling crosstalk in plants has been recognised for several decades. For example, the rice *Pigm* locus contains a cluster of genes encoding NLR receptors (*PigmR* and *PigmS*) that confer durable resistance to the fungus *Magnaporthe oryzae* without yield penalty. The transcription factor *IPA1* promotes both yield and immunity in rice via epigenetic regulation of its phosphorylation state (Wang, Zhou, et al. 2018; Wang, Wang, et al. 2018; Wang, Cheng, et al. 2018). The rice *OsALDH2B1* provides a genetic basis for growth–defence trade‐offs via its versatile functions (Ke et al. 2020). These reports exampled the crosstalk between plant defence responses and growth, whereas little is known about receptors that may be shared in this crosstalk. Here, we discovered that *RLP6* functioned both in VW defence and root biomass in *Arabidopsis* and cotton, indicating that maybe *RLP6* is a signalling intercept for both plant developmental processes and defence. A similar result was also observed in tomato, in that there were significant effects on growth and productivity in the presence and absence of a functional Ve1 protein, a gene encoding an LRR‐RLP.

To introduce the superior VW resistance trait from *G. barbadense* into *G. hirsutum* is a breeding objective for scientists. However, it is challenging due to distorted segregation, sterility, mote formation and limited recombination via traditional breeding approaches (Zhang et al. 2016). Therefore, exploring the available marker would be helpful for directly transferring target genomic segments from *G. barbadense* into *G. hirsutum* . In this study, we demonstrated that the 16 bp insertion in the *GbRLP6* promoter of *G. barbadense* is responsible for the high VW resistance difference from *G. hirsutum* , as reflected between *G. barbadense* cultivars Pima90, H7124 and 3‐79 and *G. hirsutum* cultivars NDM8, JND23 and CCRI8, indicating that this species‐diversified SV is a useful marker. This work showed that introgression of the *G. barbadense* *RLP6* allele into the *G. hirsutum* background could significantly improve the VW resistance. Thus, use of the naturally occurring *GbRLP6* allele provides a beneficial breeding strategy in *G. hirsutum* improvement.

## 4 Experimental Procedures

### 4.1 Plant Materials and Growth Conditions and VW Resistance Assay

Seeds of cottons were planted in Hoagland solution‐saturated vermiculite in an environmentally controlled greenhouse (28/25°C day/night, 16‐h photoperiod and 80% relative humidity). Plants of the wild‐type (WT) *Arabidopsis thaliana* Columbia ecotype and transgenic lines were grown at 23°C, under long‐day conditions (16 h light/8 h dark) in the greenhouse.

The severely virulent strain Linxi 2‐1 of *V. dahliae* and the *V. dahliae* with green fluorescent protein gene (Vd‐GFP) were used for inoculation. The two strains were cultured on a potato dextrose agar (PDA) for 7 days at 25°C and then cultured (120 rpm) in potato dextrose broth (PDB) at 25°C for 5–7 days. For *V. dahliae* inoculation, a total of 10 mL conidia (10^7^ conidia/mL) was injected with good distribution into the seedling pot. Symptom development was recorded at 20 and 25 dpi and categorised into five grades: 0 = healthy plant; 1 = yellowing or necrosis of 1–2 cotyledons; 2 = yellowing or necrosis of first true leaf; 3 = > 2 wilted or necrotic leaves; 4 = no leaves left or dead plant. The disease index (DI) was calculated according to the same method used in the study by Zhang et al. (2021).

### 4.2 RT‐qPCR Analysis

Total RNA was extracted from frozen samples using the EASY spin Plus Plant RNA Kit (Aidlab). cDNA was synthesised using the ReverTra Ace qPCR RT Master Mix with gDNA Remover (TOYOBO) according to the manufacturer's instructions. The *UBQ14* gene was used as the reference. The target genes were amplified with corresponding primer pairs. The primer sequences are shown in Table S1. Real‐time PCR was performed using SYBR Premix Ex Taq TM II (Perfect Real Time) in a LightCycler 96 Real‐Time PCR System (Roche). PCR cycles were as follows: 40 cycles of denaturation for 10 s at 95°C, annealling for 10 s at 60°C and extension for 15 s at 72°C. The standard curve and PCR efficiency were first analysed to optimise the comparative threshold (2^−ΔΔ*C*t^) method (Livak and Schmittgen 2000).

### 4.3 Cloning of Promoters and Sequence Analysis

Genome DNA was extracted from Pima90 and NDM8 via the method of CTAB. Primers pNHL6‐F and pNHL6‐R were used to isolate promoters. PCR was conducted as follows: 95°C for 10 min; 30 cycles of 95°C for 1 min, 56°C for 1 min and 72°C for 2 min; 72°C for 10 min; 4°C hold. The relevant *cis* elements of *RLP6* promoter were predicted using the PLACE software (http://www.dna.affrc.go.jp/htdocs/PLACE/). The primers are listed in Table S1.

### 4.4 Vector Construction, Plant Transformation and Transgenic Plant Selection

The full‐length open reading frames of *RLP6* and *NHL6* were amplified through PCR using cDNAs synthesised from RNA that was isolated from seedlings. The amplified products were further cloned into the pGreen vector driven by the CaMV 35S promoter. The resulting constructs were further transformed into *A. thaliana* Columbia type by *Agrobacterium tumefaciens* GV3101 and selected with Basta. The assay for disease resistance of transgenic *Arabidopsis* was performed according to Zhang et al. (2021). The primers used for gene cloning are listed in Table S1. The different *RLP6* promoters were inserted into vector pBI121 instead of the 35S promoter. The recombinant plasmids were transformed into *A. tumefaciens* GV3101 and then transformed into *Arabidopsis* ecotype Columbia‐0 using the floral‐dip method (Clough and Bent 1998). Transgenic lines were identified by selective medium containing kanamycin, and by PCR.

### 4.5 Histochemical Assay and Quantitative Determination of GUS

Transgenic *Arabidopsis* was cultured on ½ × Murashige and Skoog (MS) medium. The whole transgenic *Arabidopsis* and control plants were used to detect β‐glucuronidase (GUS) activity. The seedlings were immersed in reaction buffer containing 950 μL of 100 mM sodium phosphate with pH 7.2, 10 μL of 50 mM potassium ferricyanide, 10 μL of 50 mM potassium ferrocyanide, 20 μL of 1 mM X‐gluc, 10 μL of 100 mM EDTA, 1 μL of Triton X‐100 overnight. The stained samples were decolourised with 70% ethanol and observed with anatomical lens or scanner. The GUS activity of transgenic *Arabidopsis* with different promoter regions was examined quantitatively by fluorometric determination.

### 4.6 VIGS In Cotton

In VIGS experiment, the *CLA1* gene (encoding 1‐deoxyxylulose 5‐phosphate synthase) was used as the control and the specific fragments amplified from *RLP6* and *NHL6* were used to construct recombinant vectors. The recombinant vectors were transformed into *A. tumefaciens* GV3101. The empty vector was used as control. VIGS was conducted as described previously (Zhang et al. 2021). Each experimental treatment was conducted with at least 30 cotton plants.

### 4.7 RNA‐Seq Experiment and Analysis

For RNA‐Seq, 3‐week‐old seedlings of the VIGS cotton and the negative control (CK) plants were challenged with *V. dahliae* and the leaf was sampled at 12 hpi, with five plants pooled into a sample. Three biological replicates were included for each treatment. Paired‐end libraries and Illumina sequencing was performed by Novogene (Tianjin, China). *G. hirsutum* TM‐1 (https://www.cottongen.org/) was used as the reference genome to analyse the raw data with TopHat2. Then the gene expression levels were calculated based on the fragments per kilobase of transcript per million mapped reads (FPKM) values using Cuffdiff. Finally, log_2_(1 + FPKM) values were calculated after averaging three replicates.

### 4.8 *RLP6* Genetic Transformation and Generation of Transgenic Cotton Plants

The constructs pCamE‐*RLP6* under the 35S promoter control was introduced into *A. tumefaciens* GV3101 and transformed into the wild‐type cotton line (CCRI 8) according to previous research (Liu et al. 2011). After obtaining regenerated plants, the leaf DNA of these transformants was extracted and taken as template for PCR amplification using primers VPF‐4/VPR‐5. The positive transformants were identified by Sanger sequencing and PCR genotyping from the T_0_ to T_3_ generation. The T_3_ generation transgenic lines were used for function analysis.

### 4.9 Measurement of SA, Lignin and H_2_O_2_ Content

SA contents of cotton leaves were performed according to Mo et al. (2021). Measurement of lignin was performed based on the method as previously described (Zhang et al. 2017). H_2_O_2_ content was determined using the hydrogen peroxide assay kit (Nanjing Jiancheng Bioengineering Institute) according to the manufacturer's instruction.

### 4.10 Bait Recombinant Vector Construction, Identification and Y2H Library Screening

RLP6 structure was analysed with SignalP v. 3.0 (http://www.cbs.dtu.dk/services/SignalP‐3.0) and TMHMM v. 2.0 (http://www.cbs.dtu.dk/services/TMHMM‐2.0/). pGBKT7 was used to construct the bait recombinant vector with the intracellular domain of RLP6 (RLP6IN). Methods of culturing yeast and lithium acetate‐mediated yeast transformation were adopted as per the description in the Yeast Protocols Handbook (Clontech). The procedure for testing bait for auto‐activation and toxicity and Y2H library screening was operated under the guidance of Gold Yeast Two‐Hybrid System User Manual (Clontech). Bait yeast was mated with the Y2H cDNA library derived from roots of Pima90‐53 inoculated with *V. dahliae* (Yang, Ling, et al. 2014), and the positive interactions were identified on QDO/X/A agar plates.

### 4.11 Bimolecular Fluorescence Complementation

Bimolecular fluorescence complementation (BiFC) is based on the association of fluorescent protein fragments that are attached to components of the same macromolecular complex (Kerppola 2008). p326YFPN and p326YFPC vectors were granted by the Dr. Hui Du of Hebei Agricultural University (Yang et al. 2022). *RLP6* and *NHL6* were cloned into the binary BiFC vectors. The fusion constructs were also transformed into onion epidermis cells together, and then the fluorescence signal was imaged using a fluorescence microscope (Olympus). *Nodulin‐35* and *AtTRX‐o1* were used as the positive control (Yang et al. 2022). RLP6IN‐YFPN and the C‐terminal halves of YFP were taken as the negative control.

## Conflicts of Interest

The authors declare no conflicts of interest.

## Supporting information




**Figure S1.** RLP6 promoter polymorphism is associated with Verticillium wilt resistance.

**Figure S2.** PCR detection and sequencing of T_2_ transgenic cotton plants. (a) pCameE‐*RLP6* detected in transgenic cotton by PCR amplified with cross vector primers VPF‐4/VPR‐5. M: DL2000; 1–11: the tested plants; 12: blank control; 13: negative control; 14: positive control. The 750 bp fragment was amplified. (b) Sequence analysis of PCR products from the tested plants. Blue box indicated the start overlapping sequences of PCR products and pCameE vector. Red box indicated translation initiation codon ATG, the about 200 bp sequence in front of ATG was a part of pCameE vector. The results showed that *RLP6* gene was successfully transformed into the genome of upland cotton CCRI8.

**Figure S3.** Testing and identification of the bait yeast pGBKT7‐RLP6IN (Y2HGold). (a) Testing bait for toxicity. Y2HGold yeast with empty vector pGBKT7 was taken as the control. (b) Testing bait for autoactivation. It is imperative to confirm that the bait does not autonomously activate the reporter genes in Y2HGold, in the absence of a prey protein. The diploid Y2HGold (pGBKT7‐53) and Y187 (pGADT7‐T) were taken as positive control, and the diploid Y2HGold (pGBKT7‐Lam) and Y187 (pGADT7‐T) were taken as negative control.

**Figure S4.** Detection of positive *Escherichia coli* with potential genes interacting with RLP6. (a) Proteins screening interacting with RLP6 based on yeast two‐hybrid screening. (b) PCR products of potential genes interacting with RLP6. (c) Detection of positive *E. coli* with potential genes interacting with RLP6. (d, e) Autoactivation testing of prey yeast.

**Figure S5.** The cooperation between RLP6 and NHL6. (a) The RLP6‐NHL6‐silenced cotton displayed more serious symptoms of Verticillium wilt. (b) Expression of *NHL6* in *RLP6*‐silenced cotton, and the expression of *RLP6* in *NHL6*‐silenced cotton.

**Figure S6.** Phylogenetic analysis of RLP6 protein in cotton.

**Table S1.** The primers used in this study.

## Data Availability

All the authors confirm that the data supporting the findings of this study are available within the article and its Supporting Information.
