# Supplementary figures and images for: Cotton RLP6 Interacts With NDR1/HIN6 to Enhance Verticillium Wilt Resistance via Altering ROS and SA
Source: Mol Plant Pathol. 2025 Jan 22;26(1):e70052. doi: 10.1111/mpp.70052 (PMC11753439; doi:10.1111/mpp.70052)

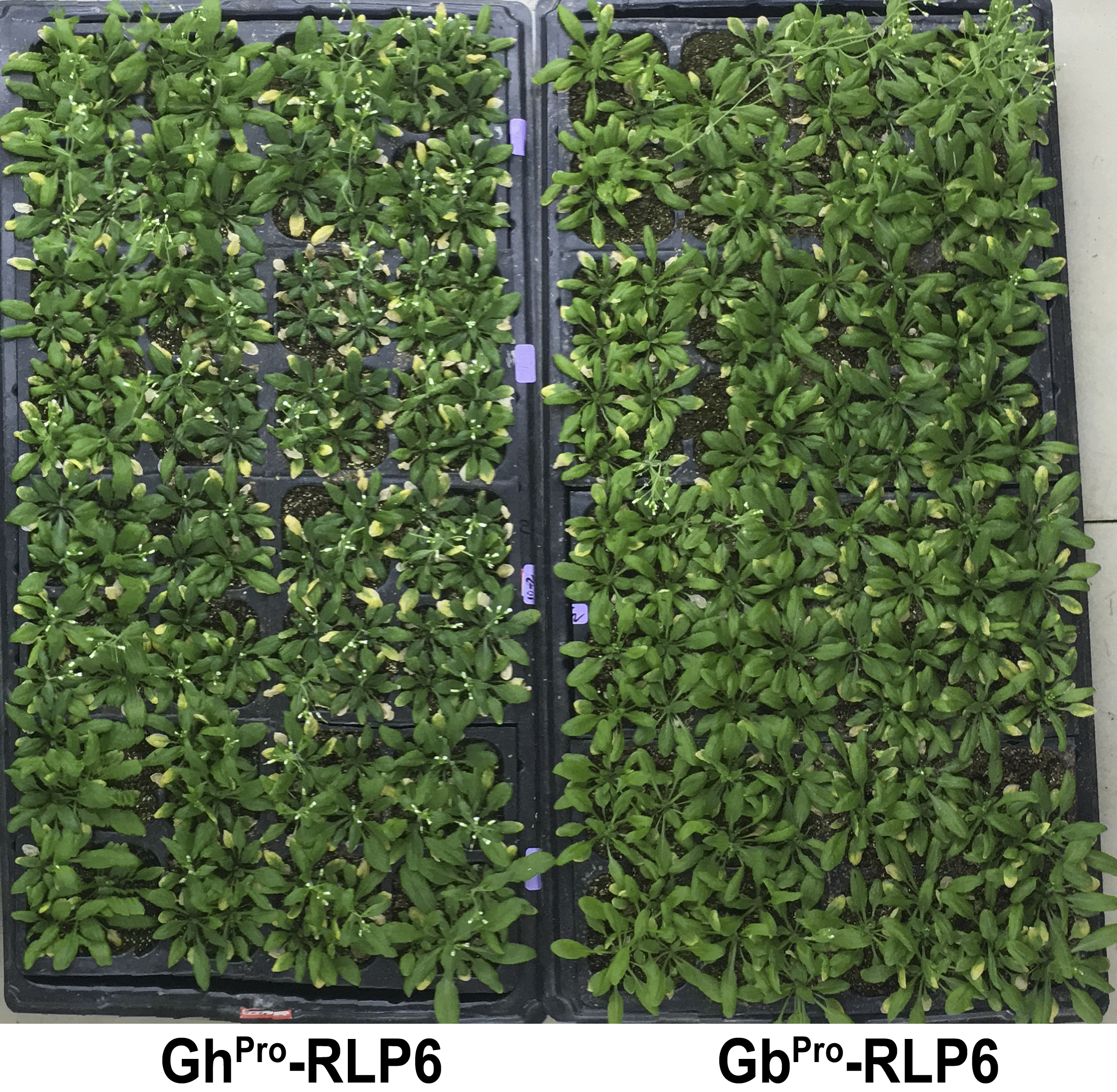

Supplement: Supplementary file 1 — Figure S1. RLP6 promoter polymorphism is associated with Verticillium wilt resistance. [file MPP-26-e70052-s002.jpg]

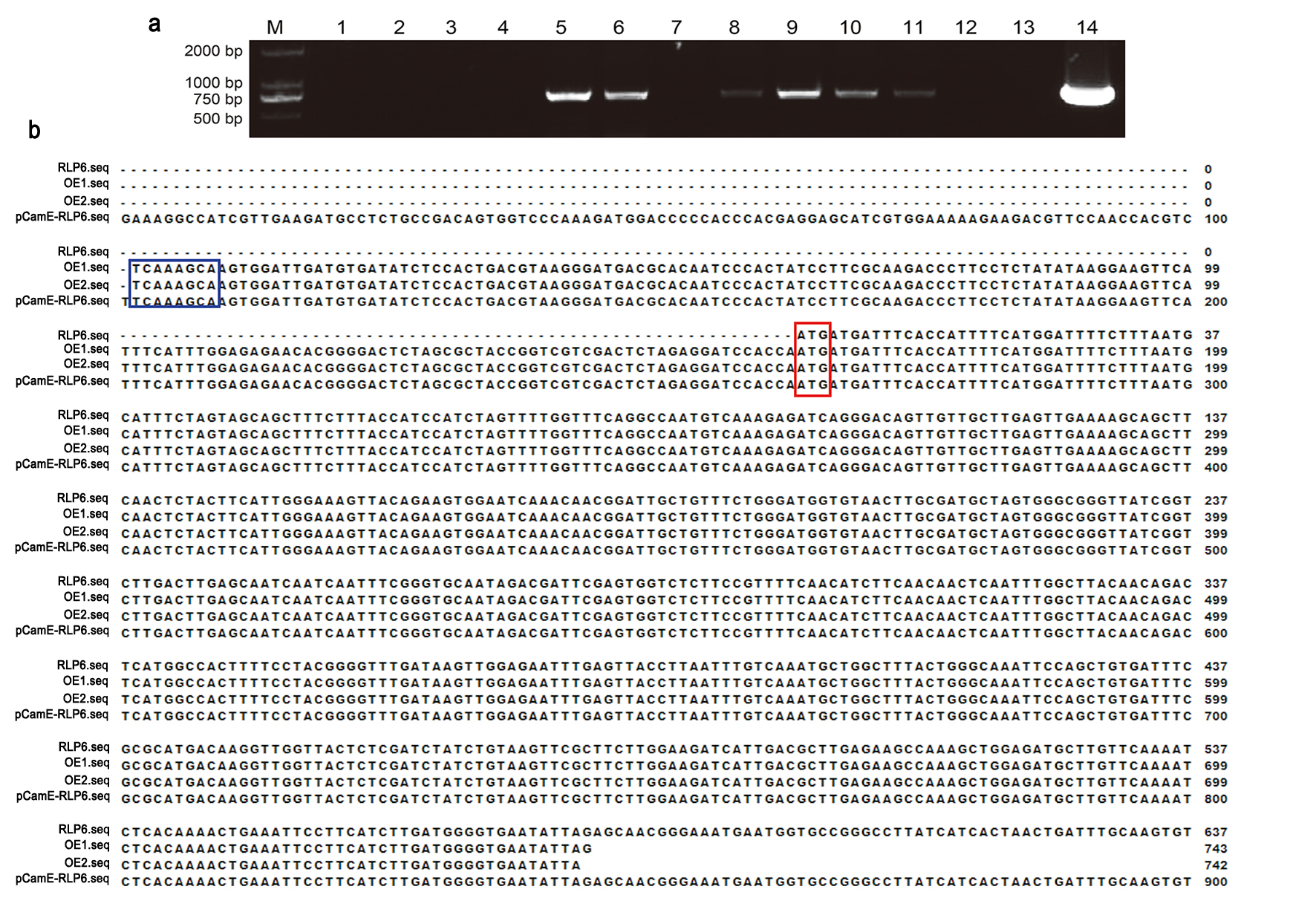

Supplement: Supplementary file 2 — Figure S2. PCR detection and sequencing of T2 transgenic cotton plants. (a) pCameE‐RLP6 detected in transgenic cotton by PCR amplified with cross vector primers VPF‐4/VPR‐5. M: DL2000; 1–11: the tested plants; 12: blank control; 13: negative control; 14: positive control. The 750 bp fragment was amplified. (b) Sequence analysis of PCR products from the tested plants. Blue box indicated the start overlapping sequences of PCR products and pCameE vector. Red box indicated translation initiation codon ATG, the about 200 bp sequence in front of ATG was a part of pCameE vector. The results showed that RLP6 gene was successfully transformed into the genome of upland cotton CCRI8. [file MPP-26-e70052-s001.jpg]

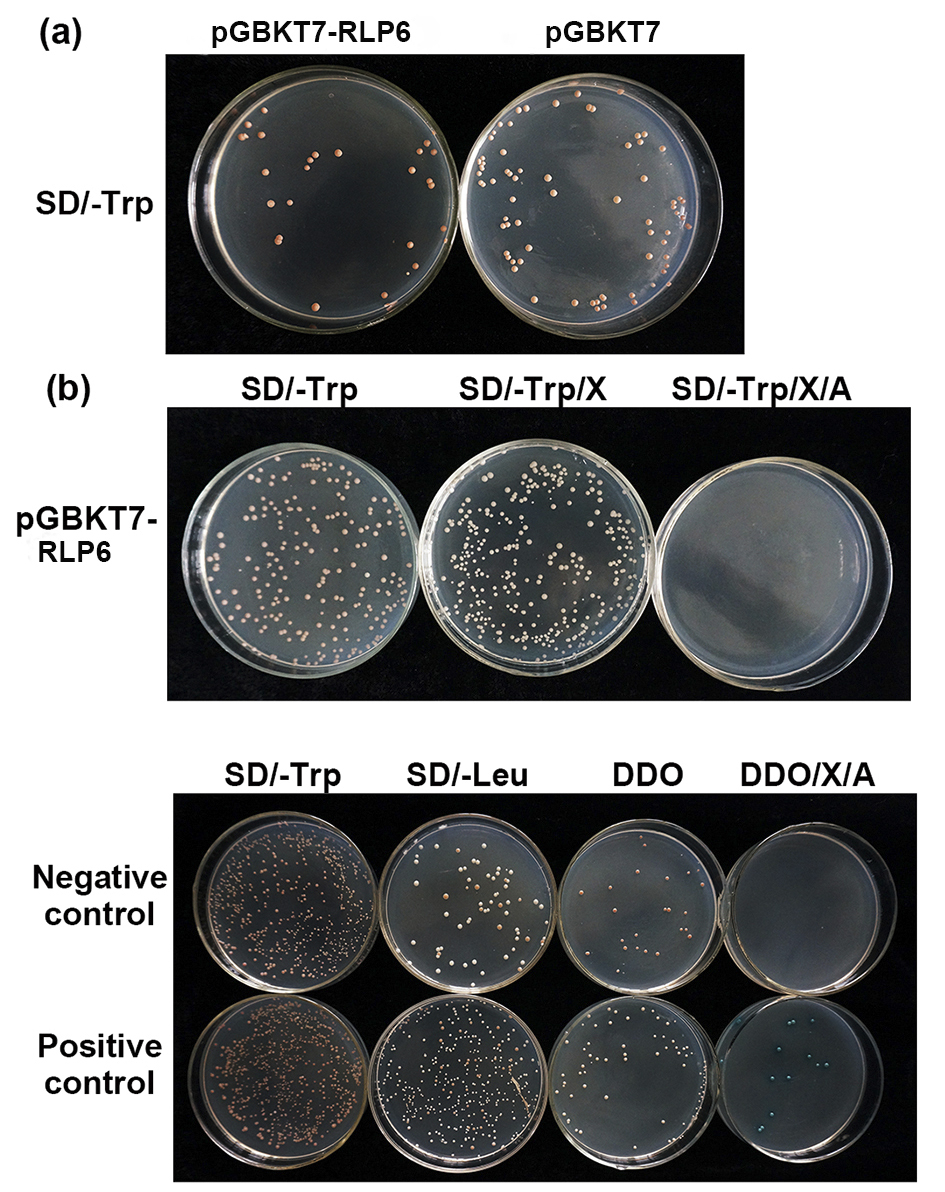

Supplement: Supplementary file 3 — Figure S3. Testing and identification of the bait yeast pGBKT7‐RLP6IN (Y2HGold). (a) Testing bait for toxicity. Y2HGold yeast with empty vector pGBKT7 was taken as the control. (b) Testing bait for autoactivation. It is imperative to confirm that the bait does not autonomously activate the reporter genes in Y2HGold, in the absence of a prey protein. The diploid Y2HGold (pGBKT7‐53) and Y187 (pGADT7‐T) were taken as positive control, and the diploid Y2HGold (pGBKT7‐Lam) and Y187 (pGADT7‐T) were taken as negative control. [file MPP-26-e70052-s004.jpg]

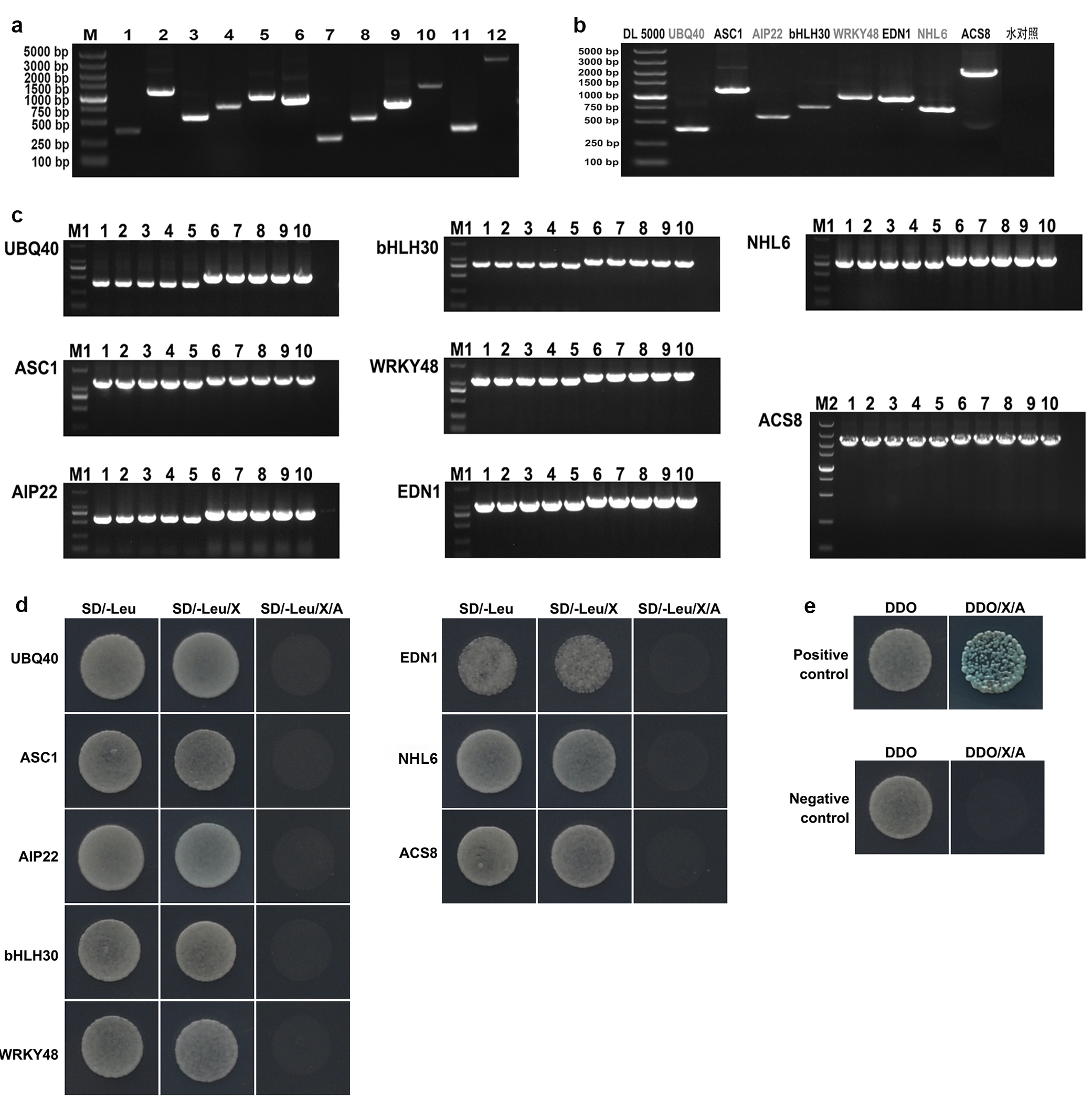

Supplement: Supplementary file 4 — Figure S4. Detection of positive Escherichia coli with potential genes interacting with RLP6. (a) Proteins screening interacting with RLP6 based on yeast two‐hybrid screening. (b) PCR products of potential genes interacting with RLP6. (c) Detection of positive E. coli with potential genes interacting with RLP6. (d, e) Autoactivation testing of prey yeast. [file MPP-26-e70052-s003.jpg]

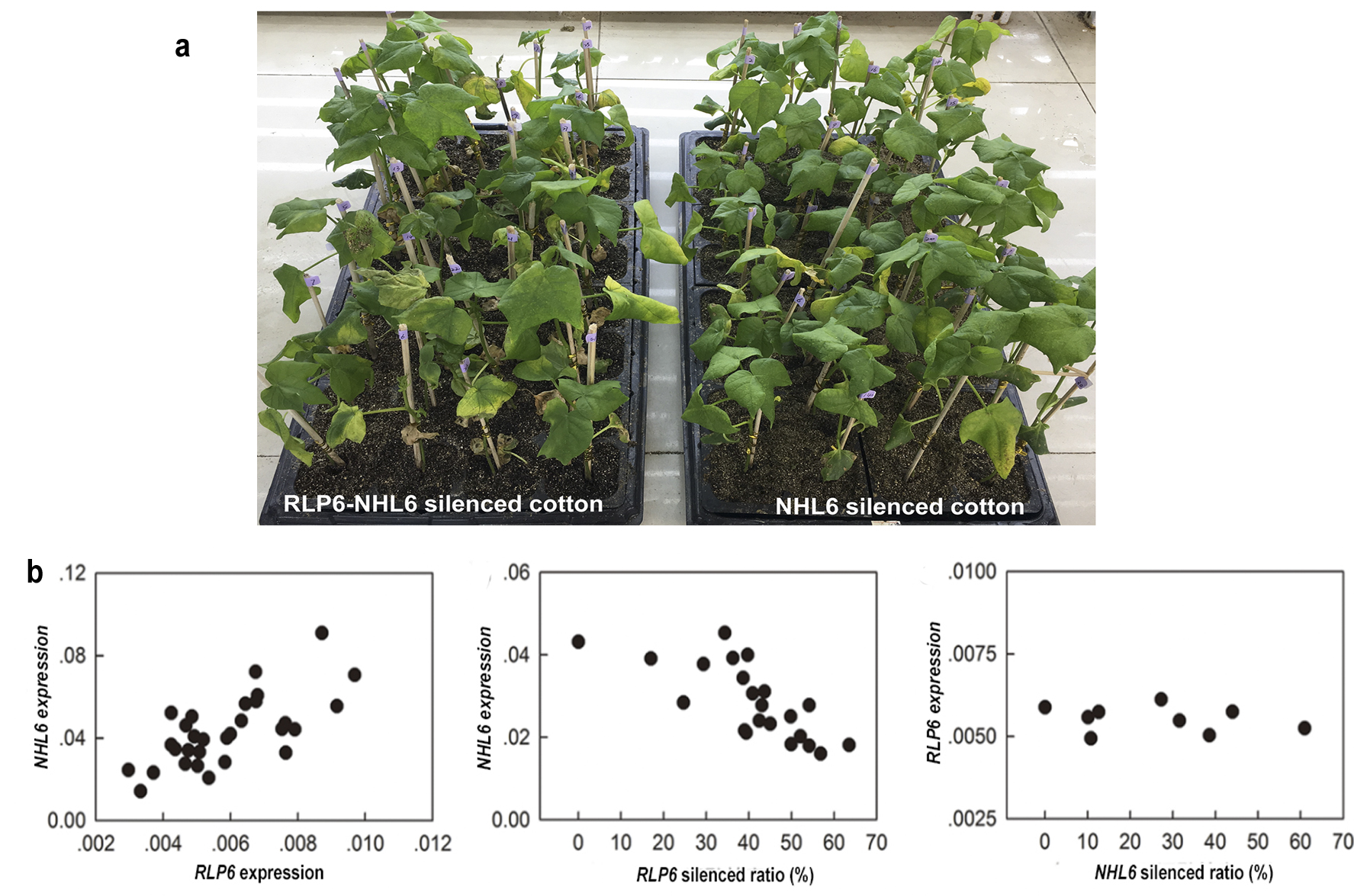

Supplement: Supplementary file 5 — Figure S5. The cooperation between RLP6 and NHL6. (a) The RLP6‐NHL6‐silenced cotton displayed more serious symptoms of Verticillium wilt. (b) Expression of NHL6 in RLP6‐silenced cotton, and the expression of RLP6 in NHL6‐silenced cotton. [file MPP-26-e70052-s006.jpg]

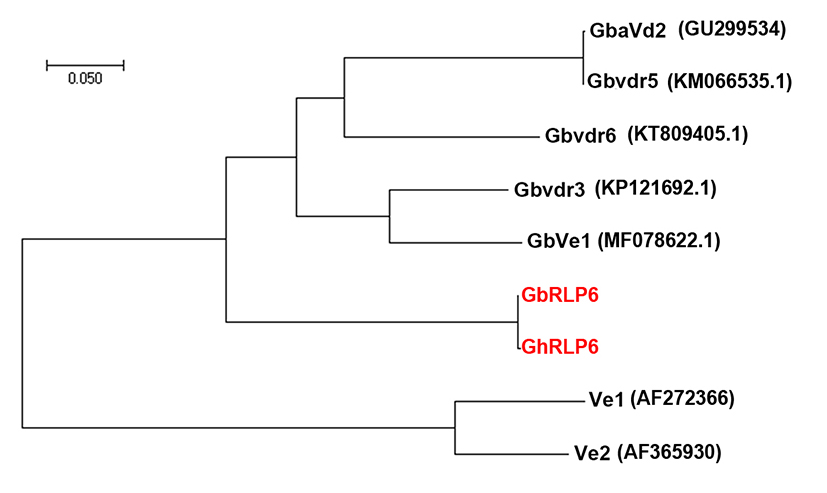

Supplement: Supplementary file 6 — Figure S6. Phylogenetic analysis of RLP6 protein in cotton. [file MPP-26-e70052-s007.jpg]
