# Supplementary material for: Cotton RLP6 Interacts With NDR1/HIN6 to Enhance Verticillium Wilt Resistance via Altering ROS and SA
Source: Mol Plant Pathol. 2025 Jan 22;26(1):e70052. doi: 10.1111/mpp.70052 (PMC11753439; doi:10.1111/mpp.70052)
Supplement: Supplementary file 7 — Table S1. The primers used in this study. [file MPP-26-e70052-s005.docx]

Table S1 Primers used in this study

| **Primer name** | **Sequence (5’-3’)** | **Purpose** |
| --- | --- | --- |
| orfRLP6-F | ATGATGATTTCACCATTTTCATGG | RLP cloning |
| orfRLP6-F | CTAAGGAGAAAAGGGAGGAGAGGTT |  |
| VPF4 | CAAAGCAAGTGGATTGATGTGATA | Detection of transgenic cotton |
| VPR5 | CGTTGCTCTAATATTCACCCCAT |  |
| pNHL6-F | GAGTAATTGAATTGAAACCAGAGCG | Clone *RLP6* promoter |
| pNHL6-R | TAAGAGTGGGAATAGGTTGAGGCAT |  |
| qNHL6-F | CGATGCTAGTGGGCGGGTTAT | qRT-PCR for *NHL6* |
| qNHL6-R | TTCTCCAACTTATCAAACCCCGTAG |  |
| EDS1-F | AATGAAGATTGATGGGTCGGAGTTAC | qRT-PCR for *EDS1* |
| EDS1-R | CCTCAGTTTGAAGCGAAGATTTAGCC |  |
| NDR1-F | CCCGTAACCAAGGAGGCTGT | qRT-PCR for *NDR1* |
| NDR1-R | CTGCTAAGGGAAGGCAAGGATAG |  |
| NPR1-F | GTCTGGCTGATGTCAATCTGCG | qRT-PCR for *NPR1* |
| NPR1-R | TCCTTCCCTTGCTCTGTCTTGG |  |
| ICS1-F | ATGGATGAATGGGTGCGAAGG | qRT-PCR for *ICS1* |
| ICS1-R | AAGAATGCCAGAGGTAAGAGGAGGA |  |
| PR1-F | AGACTACCTCAACGCTCACAACAC | qRT-PCR for *PR1* |
| PR1-R | CAACCCACATATTTACAGCATCG |  |
| PR4-F | CGGGGTGTTCCTTGATGCTC | qRT-PCR for *PR4* |
| PR4-R | TTACGCTCCACCGTCCTCTTC |  |
| PR5-F | GCCAGGGATTCTATCAAACGC | qRT-PCR for *PR5* |
| PR5-R | ATCCTCGGAGCAATGGGTTC |  |
| NHL6-F | GCGATCAAGGGATTCAACCTCACG | qRT-PCR for *NHL13* |
| NHL6-R | GCACGGCACCGTTCCTTTATTTTG |  |
| UBQ14-F | CAACGCTCCATCTTGTCCTT | The reference gene *UBQ14* |
| UBQ14-R | TAGTCGTCTTTCCCGTAAGC |  |
| M13-F | CGCCAGGGTTTTCCCAGTCACGAC | Universal primer for pMD19-T vector |
| M13-R | GAGCGGATAACAATTTCACACAGG |  |
| RLP6-318-F | CCATATGTTTATGAAGACAGCAAGTAAATGGGT | Amplification of the *NHL6* intracellular |
| RLP6-318-R | GGATCCAGGAGAAAAGGGAGGAGAGGTTGA |  |
| BD-F | TTTGTAATACGACTCACTATAGGGCG | Universal primer for pGBKT7 vector |
| BD-R | TTTTCGTTTTAAAACCTAAGAGTCAC |  |
| AD-F | TAATACGACTCACTATAGGGCG | Universal primer for pGADT7 vector |
| AD-R | AGATGGTGCACGATGCACAG |  |
| UBQ-F | TTCCATATGGAAATGCAGATATTCGTGAAAACCC | Cloning of *UBQ* gene |
| UBQ-R | CGCGGATCCCTACTTGATCTTCTTCTTCGGCC |  |
| ASC1-F | TGAAGCACAAGAGAGGAAAATAATG | Cloning of *ASC1* gene |
| ASC1-R | TCAAGTTGCTTCTATCTTTTTATCG |  |
| AIP22-F | CATATGCAGTGGAATTAAGGGTATGGC | Cloning of *AIP22* gene |
| AIP22-R | TCAAACAACACAACCCAAGCCTC |  |
| bHLH30-F | CATATGTTATTTTTGTGTGTGTTTTTATATG | Cloning of *bHLH30* gene |
| bHLH30-R | GAGCTCGAAACAATGGCAATCATCTTTTTTA |  |
| WRKY48-F | CATATGTCGTTGTTTTTCGTGAAAATG | Cloning of *WRKY48* gene |
| WRKY48-R | GAGCTCGCGTTTATTTGTTTATCAAGT |  |
| EDN1-F | CCCGGGCACATCGATAAATAAAAAATG | Cloning of *EDN1* gene |
| EDN1-R | GAGCTCTCATTTGAGCCAAAAAAGCTA |  |
| NHL6-F | CATATGGAAATGGCCGAACGAATCCACCCCG | Cloning of *NHL13* gene |
| NHL6-R | CGAGCTCTTACCAAAGATCTACTCCATAATCA |  |
| ACS8-F | CCCGGGTCAATATAAACATTCAGAATG | Cloning of *ACS8* gene |
| ACS8-R | GAGCTCGCATTTATTATGGGTATATTA |  |
| UBQAD-F | CATATGGAAATGCAGATATTCGTGAAAACCCT | pGADT7-*UBQ* construction |
| UBQAD-R | CGAGCTCCTTGATCTTCTTCTTCGGC |  |
| ASC1AD-F | CATATGATGGAGACGGCAGGAGAATGGC | pGADT7-*ASC1* construction |
| ASC1AD-R | GAGCTCAGTTGCTTCTATCTTTTTATCGGTC |  |
| AIP22AD-F | GCGCATATGATGGCATTTGAGAAAGATC | pGADT7-*AIP22* construction |
| AIP22AD-R | GGATCCAACAACACAACCCAAGCCTCT |  |
| bHLH30AD-F | CATATGATGGCTGCTTTCTATTTCAACA | pGADT7-*bHLH30* construction |
| bHLH30AD-R | GAGCTCTTATGAAGATGAGCTTGAGGAATCA |  |
| WRKY48AD-F | CATATGGAAATGGCGGTGGATCTGATGAGT | pGADT7-*WRKY48* construction |
| WRKY48AD-R | GAGCTCAGTTGACTTGAATACTACCAG |  |
| EDN1AD-F | GAATTCATGCAGTGTGGAAGGCAAGCAGGTG | pGADT7-*EDN1* construction |
| EDN1AD-R | GAGCTCCATTGAGTCCACCGAGACTCCATTC |  |
| NHL6IN-F | CATATGCGCATGGCCGAACGAATCCACC | pGADT7-*NHL13IN* construction |
| NHL6IN-R | CGAGCTCAAAGCAGCAGCAGCAGCGGCAAC |  |
| NHL6OU-F | CATATGCGCATGAAGCCCGAAGCTCCAAACTAC | pGADT7-*NHL13OU* construction |
| NHL6OU-R | CGAGCTCCCAAAGATCTACTCCATAATCAC |  |
| ACS8IN-F | CATATGATGAGAAAGAAAAAGGGGAAAAAAAGAG | pGADT7-*ACS8IN* construction |
| ACS8IN-R | GAGCTCAACTCCCTTTGGCAGACCTGTACT |  |
| ACS8OU-F | CATATGATGAATGATGTATACTTGGCATAT | pGADT7-*ACS8OU* construction |
| ACS8OU-R | GAGCTCCTGGTACAACTTTTGGAGATCATCT |  |
| RLP6INYFP-F | TCTAGAATGAAGACAGCAAGTAAATGGGT | RLP6IN-YFPN construction |
| RLP6INYFP-R | ACTAGTAGGAGAAAAGGGAGGAGAGGTT |  |
| NHL6INYFP-F | TCTAGAATGGCCGAACGAATCCACCCCG | NHL6-YFPC construction |
| NHL6INYFP-R | ACTAGTAAAGCAGCAGCAGCAGCGGCAAC |  |
| 35s-F | GACGCACAATCCCACTATCCTTCGCA | Universal primer for BiFC vector |
| HA-R | CCGCTCCCGCATAGTCAGGAACATC |  |
| RLP6V-F | GTCGACATGGGGACTCACATCTTGAAATC | Gene silenced primer for *RLP6* |
| RLP6V-R | GGTACCGCGCAAGATGTTTTGGAATA |  |
| NHL6V-F | TCTAGATTTTAACAACTATCCTCTCTCTCC | Gene silenced primer for *NHL6* |
| NHL6V-R | GAGCTCTCCTTGGATACAATCTTGGACGC |  |
| RLP6-Indel-F | AATTAGATTAAAGTATTTATAACAT | Genotyping based on 16bp indel |
| RLP6-Indel-R | ATTGTTGCGTTTGTTTTACGGCTT |  |
